# Association of nineteen polymorphisms from seven DNA repair genes and the risk for bladder cancer in Gansu province of China

**DOI:** 10.18632/oncotarget.9146

**Published:** 2016-05-02

**Authors:** Gongjian Zhu, Haixiang Su, Lingeng Lu, Hongyun Guo, Zhaohui Chen, Zhen Sun, Ruixia Song, Xiaomin Wang, Haining Li, Zhiping Wang

**Affiliations:** ^1^ School of Life Sciences, Lanzhou University, Lanzhou, Gansu 730000, China; ^2^ Institute of Urology, the Second Hospital of Lanzhou University, Lanzhou, Gansu 730000, China; ^3^ Gansu Provincial Academy of Medical Sciences, Gansu Provincial Cancer Hospital, Lanzhou, Gansu 730050, China; ^4^ Department of Chronic Disease Epidemiology, Yale School of Public Health, School of Medicine, Yale Cancer Center, Yale University, New Haven, CT 06520-8034, USA; ^5^ Department of Internal Medicine, Xigu District of Lanzhou City People's Hospital, Lanzhou, Gansu 730050, China

**Keywords:** bladder cancer, polymorphisms, DNA repair, gene interaction

## Abstract

**Background:**

Balance of DNA damage and proper repair plays an important role in progression of bladder cancer. Here we aimed to assess the associations of nineteen polymorphisms from seven DNA repair–associated genes (*PRAP1, OGG1, APEX1, MUTYH, XRCC1, XRCC2* and *XRCC3*) with bladder cancer and their interactions in the disease in a Han Chinese population.

**Methodology/Principal Findings:**

A chip-based TaqMan genotyping for the candidate genes was performed on 227 bladder cancer patients and 260 healthy controls. *APEX1* rs3136817, *MUTYH* rs3219493, three SNPs (rs3213356, rs25487 and rs1799782) in *XRCC1*, and three SNPs (rs1799794, rs861531 and rs861530) in *XRCC3* showed significant associations with the risk of bladder cancer. In haplotype analysis, elevated risks of bladder cancer were observed in those with either haplotype GT (OR = 1.56, *P* = 0.003) of *APEX1*, or GGGTC (OR = 2.05, *P* = 0.002) of *XRCC1*, whereas decreased risks were in individuals with either GCGCC (OR = 0.40, *P* = 0.001), or GCGTT (OR = 0.60, = 0.005) of *XRCC1*, or CCC (OR = 0.65, *P* = 0.004) of *MUTYH*, or TTTAT (OR = 0.36, *P* = 0.009) of *XRCC3*. Interaction analysis showed that the two-loci model (rs1799794 and rs861530) was the best with the maximal testing accuracy of 0.701, and the maximal 100% cross-validation consistency (*P* = 0.001).

**Conclusions:**

Polymorphisms and haplotypes of DNA repair genes are associated with the risk of bladder cancer, and of which the SNPs (rs1799794 and rs861530) in *XRCC3* gene might be two major loci in relation to the susceptibility to bladder cancer in a northwest Chinese population.

## INTRODUCTION

Bladder cancer is the most frequent malignancy of urinary tract cancer and the fourth most incident cancer in males and the seventh most incident in females in the world [1]. Epidemiological data provided by the International Agency for Research on Cancer (IARC) in 2012 show 55,486 cases and 26,820 deaths of bladder cancer in China [2], of which over 90% were Han Chinese. Risk factors for bladder tumorigenesis include genetic and molecular abnormalities, chemical or environmental exposures, and chronic irritation [3]. Deficiency of DNA damage repair systems is thought as one of the mechanisms underlying carcinogens- and mutagens-induced tumors [4]. There are two major types of DNA damage, in which different repair pathways are involved. The base excision repair (BER) for single-strand DNA breaks, which is in charge of removing oxidized DNA bases [5]. Homologous recombination repair (HRR) and non-homologous end joining (NHEJ) are two principle mechanisms in double-strand DNA breaks repair [6]. Accumulating evidence indicates that a reduced DNA damage repair capacity can lead to a predisposition to accumulated DNA damage, mutations, and subsequently developing diseases such as cancer [7]. Genetic polymorphisms at one or more loci in DNA repair genes are associated with DNA repair functions, and thus, may modify the impact of environmental exposures on cancer risk [8, 9].

Because of the nature of the bladder as an important void organ, the urothelial cells are continuously exposed to many DNA-damaging compounds via the filtration into urine [10]. Given that the importance of DNA repair genes in preventing potential mutation accumulation, and that genetic polymorphisms affect gene activities via altering RNA/DNA secondary structures or its encoded proteins [11, 12] it has been postulated that genetic variation may modify bladder cancer risk [13]. To our knowledge, however, the published studies assessing the relationship between DNA repair genes and bladder cancer risk in the Chinese population mainly focus on a single gene or a single polymorphism [14, 15]. Given that multiple genes are involved in DNA damage repair systems, we ask whether there is any synergistic effects between SNPs of these genes on the risk of bladder cancer. Thus, we conducted this case-control study to investigate the associations between nineteen polymorphisms from five genes (*PARP1*, *OGG1*, *APEX1*, *MUTYH*, *and XRCC1*) of BER and two genes (*XRCC2 and XRCC3*) of HRR, which were chosen based on the literature on other types of human cancer [16–18], and the risk of bladder cancer in Gansu Province of China.

## RESULTS

### Baseline characteristics

Details of the study population are shown in Table 1. The mean ages of the bladder cancer patients and the controls were 54.6 ± 7.8 and 53.8 ± 8.4 years, respectively. No significant differences in age (*P* = 0.506), gender (*P* = 0.542), cigarette smoking (*P* = 0.412) and alcohol consumption (*P* = 0.116) were observed between the patients and the controls.

**Table 1:** Population characteristics

Variable	Case (%)	Control (%)	*P* value
Total number of patients	227	260	
Gender			0.506^a^
Female	40 (17.62)	40 (15.38)	
Male	187 (82.38)	220 (84.62)	
Reference age, years			0.542
< 40	11 (4.85)	10 (3.84)	
40–49	30 (13.22)	45 (17.31)	
50–59	50 (22.03)	66 (25.38)	
60–69	72 (31.72)	75 (28.85)	
≥ 70	64 (28.19)	64 (24.62)	
Stage			
Non-invasive	159 (70.04)		
Invasive	68 (29.96)		
Size, cm			
< 3	137 (60.35)		
≥ 3	90 (39.65)		
Multiplicity			
Single	138 (60.79)		
Multiple	89 (39.21)		
Pathological classification			
G1	59 (25.99)		
G2	98 (43.17)		
G3	80 (35.24)		
Smoking status			0.412
Yes	80 (35.24)	101 (38.85)	
No	147 (64.76)	159 (61.15)	
Alcohol consumpation			0.116
Yes	76 (33.48)	105 (40.38)	
No	151 (66.52)	155 (59.61)	

a For chi-square test (two-side).

### Nineteen polymorphisms and the risk of bladder cancer

The associations between DNA repair gene polymorphisms and bladder cancer risk are shown in Table 2. No deviation from Hardy–Weinberg equilibrium was found in the genotype frequencies for all nineteen SNPs in the control subjects (*P* > 0.05). In *APEX1* rs3136817, compared to the TT genotype and T allele, TC genotype and C allele were associated with a decreased risk of bladder cancer (*P* = 0.002, adjusted OR = 0.48, 95% CI: 0.30–0.77; *P* = 0.005, adjusted OR = 0.56, 95% CI: 0.38-0.84, respectively). Additionally, *APEX1* rs3136817 conferred a decreased risk of bladder cancer in dominant model (CC + TC vs. TT, *P* = 0.002, adjusted OR = 0.49, 95% CI: 0.31–0.77). In *MUTYH* rs3219493, compared to the C allele, G allele was associated with an increased risk of bladder cancer (*P* = 0.002, adjusted OR = 1.80, 95% CI: 1.24–2.61). In *XRCC1* rs3213356, compared to TT genotype, CT genotype showed a decreased risk while CC genotype showed an increased risk of bladder cancer (*P* = 0.002, adjusted OR = 0.43, 95% CI: 0.25–0.74; *P* = 0.005, adjusted OR = 5.76, 95% CI: 1.69–19.67, respectively). The effect of *XRCC1* rs3213356 exhibited significant in the recessive model (*P* = 0.002, adjusted OR = 6.87, 95% CI: 2.03–23.04) but not in the dominant one. In *X* RCC1 rs25487, compared to the CC genotype, CT genotype was associated with a decreased risk of bladder cancer (*P* = 0.002, adjusted OR = 0.48, 95% CI: 0.31–0.76). Moreover, rs25487 conferred a decreased risk of bladder cancer in the dominant model (TT + CT vs. CC, *P* = 0.009, adjusted OR = 0.58, 95% CI: 0.38–0.87). In *XRCC1* rs1799782, compared to the GG genotype, AA genotype was associated with an increased risk of bladder cancer (*P* = 0.005, adjusted OR = 2.67, 95% CI: 1.35–5.26), and this SNP was also associated with an increased risk under recessive model (AA vs. GG + GA, *P* = 0.001, adjusted OR = 2.91, 95% CI: 1.51–5.61). In *XR* CC3 rs1799794, compared to the TT genotype, TC genotype showed an association with decreased risk of bladder cancer (*P* = 0.001, OR = 0.33, 95% CI: 0.20–0.55). Furthermore, the dominant model showed a decreased risk (CC + CT vs TT, *P* = 0.006, adjusted OR = 0.54, 95% CI: 0.35–0.84) while the recessive model showed an increased risk (CC vs. TT + CT, *P* = 0.002, adjusted OR = 2.12, 95% CI: 1.31–3.43) of bladder cancer. In *XRCC3* rs861531, compared to the CC genotype, AC genotype showed an association with a decreased risk of bladder cancer (*P* = 0.008, adjusted OR = 0.47, 95% CI: 0.27-0.82). *XRCC3* rs861530 CT genotype showed a decreased risk compared to TT genotype (*P* = 0.002, adjusted OR = 0.48, 95% CI: 0.30–0.78), and this SNP was associated with an increased risk under recessive model (CC vs. TT + CT, *P* = 0.001, adjusted OR = 2.19, 95% CI: 1.35–3.55) of bladder cancer. After FDR correction for multiple testing, these associations were still significant (*Q*_FDR_ < 0.05). However, no association with the risk of bladder cancer was observed for other SNPs in these DNA repair genes after FDR correction.

**Table 2:** Genotype frequencies of gene polymorphisms in controls and cases and their associations with bladder cancer

Gene	SNP	Genotype	Case *N* (%)	Control *N* (%)	Adj-OR (95% CI)^a^	*P* value	*Q*_FDR_^b^
PARP1		CC	43 (29.86)	75 (28.96)	Reference		
	rs1805415	CT	70 (48.61)	136 (52.51)	0.82 (0.49–1.36)	0.441	0.635
	synonymous	TT	31 (21.53)	48 (18.53)	0.98 (0.52–1.84)	0.946	1.010
		C	156 (54.17)	287 (55.30)	Reference		
		T	132 (45.83)	232 (44.70)	0.97 (0.71–1.33)	0.863	0.965
		Dominant^c^			0.86 (0.53–1.39)	0.543	0.697
		Recessive^d^			1.11 (0.65–1.91)	0.701	0.843
*P* value for HWE		0.378					
OGG1		GG	63 (28.38)	92 (35.38)	Reference		
	rs2072668	CG	116 (52.25)	117 (45.00)	1.42 (0.91–2.21)	0.127	0.262
	intron	CC	43 (19.37)	51 (19.62)	1.34 (0.76–2.35)	0.317	0.486
		G	242 (54.50)	301 (57.88)	Reference		
		C	202 (45.50)	219 (42.12)	1.19 (0.90–1.57)	0.230	0.412
		Dominant			1.39 (0.91–2.12)	0.123	0.260
		Recessive			1.08 (0.66–1.78)	0.758	0.889
*P* value for HWE		0.214					
APEX1		TT	157 (77.72)	165 (63.71)	Reference		
	rs3136817	TC	39 (19.31)	88 (33.98)	**0.48 (0.30–0.77)**	**0.002**	**0.019**
	intron	CC	6 (2.97)	6 (2.32)	0.64 (0.18–2.22)	0.480	0.661
		T	353 (87.38)	418 (80.69)	Reference		
		**C**	51 (12.62)	100 (19.31)	**0.56 (0.38–0.84)**	**0.005**	**0.026**
		Dominant			**0.49 (0.31–0.77)**	**0.002**	**0.019**
		Recessive			0.78 (0.23–2.68)	0.689	0.850
*P* value for HWE		0.142					
	rs1130409	TT	51 (29.82)	78 (30.12)	Reference		
	missense	GT	71 (41.52)	130 (50.19)	0.83 (0.51–1.35)	0.448	0.635
		GG	49 (28.65)	51 (19.69)	1.41 (0.80–2.48)	0.241	0.424
		T	173 (50.58)	286 (55.21)	Reference		
		G	169 (49.42)	232 (44.79)	1.18 (0.88–1.58)	0.278	0.455
		Dominant			0.99 (0.63–1.56)	0.974	1.006
		Recessive			1.57 (0.97–2.56)	0.067	0.182
*P* value for HWE		0.859					
MUTYH		CC	126 (66.32)	183 (70.38)	Reference		
	rs3219493	GC	43 (22.63)	72 (27.69)	1.04 (0.64–1.69)	0.860	0.973
	intron	GG	21 (11.05)	5 (1.92)	7.39 (2.52–21.70)	**< 0.001**	< 0.100
		C	295 (77.63)	438 (84.23)	Reference		
		G	85 (22.37)	82 (15.78)	**1.80 (1.24–2.61)**	**0.002**	**0.019**
		Dominant			1.45 (0.93–2.26)	0.099	0.229
*P* value for HWE		Recessive			7.30 (2.51–21.24)	< 0.001	< 0.100
		0.468					
	rs3219476	CC	58 (29.15)	69 (26.54)	Reference		
	intron	AC	86 (43.22)	131 (50.38)	0.84 (0.52–1.37)	0.489	0.664
		AA	55 (27.64)	60 (23.08)	1.37 (0.78–2.38)	0.270	0.458
		C	202 (50.75)	269 (51.74)	Reference		
		A	196 (49.25)	251 (48.27)	1.17 (0.88–1.56)	0.275	0.458
		Dominant			0.99 (0.64–1.57)	0.992	1.013
		Recessive			1.53 (0.96–2.42)	0.075	0.188
*P* value for HWE		0.886					
	rs3219472	CC	93 (60)	132 (50.77)	Reference		
	intron	CT	38 (24.52)	106 (40.77)	0.54 (0.33–0.88)	0.013	0.051
		TT	24 (15.48)	22 (8.46)	1.68 (0.84–3.36)	0.146	0.289
		C	224 (72.26)	370 (71.15)	Reference		
		T	86 (27.74)	150 (28.85)	0.99 (0.71–1.40)	0.966	1.008
		Dominant			0.73 (0.47–1.12)	0.150	0.291
		Recessive			2.11 (1.08–4.15)	0.030	0.102
*P* value for HWE		0.912					
XRCC1		TT	143 (76.47)	183 (70.38)	Reference		
	rs3213356	CT	27 (14.44)	73 (28.08)	**0.43 (0.25–0.74)**	**0.002**	**0.019**
	intron	CC	17 (9.09)	4 (1.54)	**5.76 (1.69–19.67)**	**0.005**	**0.026**
		T	313 (83.69)	439 (84.42)	Reference		
		C	61 (16.31)	81 (15.58)	0.98 (0.65–1.46)	0.903	0.998
		Dominant			0.66 (0.41–1.07)	0.091	0.222
		Recessive			**6.87 (2.03–23.24)**	**0.002**	**0.019**
*P* value for HWE		0.276					
	rs25487	CC	125 (62.19)	128 (49.23)	Reference		
	missense	CT	51 (25.37)	106 (40.77)	0.48 (0.31–0.76)	0.002	0.019
		TT	25 (12.44)	26 (10.00)	0.97 (0.50–1.85)	0.914	0.998
		C	301 (74.88)	362 (69.62)	Reference		
		T	101 (25.12)	158 (30.38)	0.76 (0.56–1.05)	0.095	0.226
		Dominant			**0.58 (0.38–0.87)**	**0.009**	**0.037**
		Recessive			1.27 (0.68–2.38)	0.450	0.629
*P* value for HWE		0.558					
	rs2293036	GG	99 (46.26)	137 (52.69)	Reference		
	intron	GA	90 (42.06)	104 (40)	1.21 (0.80–1.84)	0.363	0.531
		AA	25 (11.68)	19 (7.31)	1.93 (0.95–3.93)	0.068	0.179
		G	288 (67.29)	378 (72.69)	Reference		
		A	140 (32.71)	142 (27.31)	1.33 (0.98–1.80)	0.069	0.177
		Dominant			1.32 (0.89–1.96)	0.165	0.314
		Recessive			1.77 (0.90–3.50)	0.100	0.226
*P* value for HWE		0.903					
	rs2023614	GG	17 (10.37)	2 (0.77)	Reference		
	intron	GC	50 (30.49)	42 (16.15)	0.15 (0.03–0.77)	0.022	0.084
		CC	97 (59.15)	216 (83.08)	0.04 (0.01–0.21)	< 0.001	< 0.100
		G	84 (25.61)	46 (8.85)	Reference		
		C	244 (74.39)	474 (91.15)	0.23 (0.15–0.35)	< 0.001	< 0.100
		Dominant			0.06 (0.01–0.28)	< 0.001	< 0.100
		Recessive			0.22 (0.13–0.37)	< 0.001	< 0.100
*P* value for HWE		0.979					
	rs1799782	GG	103 (50.49)	136 (52.31)	Reference		
	missense	GA	67 (32.84)	106 (40.77)	0.81 (0.52–1.25)	0.341	0.514
		AA	34 (16.67)	18 (6.92)	**2.67 (1.35–5.26)**	**0.005**	**0.026**
		G	273 (66.91)	378 (72.69)	Reference		
		A	135 (33.09)	142 (27.31)	1.34 (0.98–1.82)	0.065	0.182
		Dominant			1.07 (0.72–1.59)	0.746	0.886
		Recessive			**2.91 (1.51–5.61)**	**0.001**	**0.012**
*P* value for HWE		0.188					
XRCC2		AA	146 (73)	191 (73.46)	Reference		
	rs3218408	AC	43 (21.5)	64 (24.62)	0.94 (0.58–1.52)	0.800	0.927
	intron	CC	11 (5.5)	5 (1.92)	3.26 (1.01–10.56)	0.049	0.145
		A	335 (83.75)	446 (85.77)	Reference		
		C	65 (16.25)	74 (14.23)	1.24 (0.84–1.84)	0.284	0.457
		Dominant			1.10 (0.70–1.72)	0.696	0.848
		Recessive			3.31 (1.03–10.67)	0.045	0.143
*P* value for HWE		0.893					
	rs3218454	TT	174 (89.69)	214 (82.31)	Reference		
	intron	AT	18 (9.28)	46 (17.69)	0.49 (0.27–0.91)	0.492	0.649
		AA	2 (1.03)	0 (0)	–	0.999	1.010
		T	366 (94.33)	474 (91.15)	Reference		
		A	22 (5.67)	46 (8.85)	0.63 (0.36–1.11)	0.109	0.241
		Dominant			0.55 (0.30–0.99)	0.047	0.144
		Recessive			–	0.999	1.010
*P* value for HWE		0.118					
XRCC3		TT	72 (39.13)	69 (26.54)	Reference		
	rs1799794	CT	53 (28.80)	142 (54.62)	0.33 (0.20–0.55)	0.001	0.012
	5′ UTR	CC	59 (32.07)	49 (18.85)	1.16 (0.67–2.01)	0.598	0.757
		T	197 (53.53)	280 (53.85)	Reference		
		C	171 (46.47)	240 (46.15)	1.01 (0.75–1.35)	0.957	1.010
		Dominant			**0.54 (0.35–0.84)**	**0.006**	**0.027**
		Recessive			**2.12 (1.31–3.43)**	**0.002**	**0.019**
*P* value for HWE		0.111					
	rs861537	CC	85 (40.87)	87 (33.46)	Reference		
	intron	CT	89 (42.79)	134 (51.54)	0.70 (0.45–1.08)	0.109	0.235
		TT	34 (16.35)	39 (15)	0.94 (0.52–1.70)	0.836	0.957
		C	259 (62.26)	308 (59.23)	Reference		
		T	157 (37.74)	212 (40.77)	0.90 (0.68–1.21)	0.490	0.656
		Dominant			0.75 (0.50–1.14)	0.177	0.330
		Recessive			1.14 (0.66–1.97)	0.628	0.785
*P* value for HWE		0.279					
	rs861534	CC	151 (87.28)	215 (82.69)	Reference		
	intron	CT	13 (7.51)	42 (16.15)	0.45 (0.22–0.91)	0.027	0.095
		TT	9 (5.20)	3 (1.15)	4.46 (1.11–18.00)	0.036	0.118
		C	315 (91.04)	472 (90.77)	Reference		
		T	31 (8.96)	48 (9.23)	1.02 (0.61–1.70)	0.942	1.017
		Dominant			0.73 (0.40–1.32)	0.295	0.467
		Recessive			489 (1.22–19.71)	0.026	0.095
*P* value for HWE		0.561					
	rs861531	CC	125 (76.22)	173 (66.54)	Reference		
	intron	AC	24 (14.63)	72 (27.69)	**0.47 (0.27–0.82)**	**0.008**	**0.035**
		AA	15 (9.15)	15 (5.77)	1.32 (0.57–3.04)	0.520	0.677
		C	274 (83.54)	418 (80.38)	Reference		
		A	54 (16.46)	102 (19.62)	0.79 (0.53–1.18)	0.250	0.432
		Dominant			0.62 (0.38–1.00)	0.050	0.144
		Recessive			1.56 (0.68–3.56)	0.295	0.459
*P* value for HWE		0.049					
	rs861530	TT	68 (36.96)	70 (26.92)	Reference		
	intron	CT	60 (32.61)	144 (55.38)	**0.48 (0.30–0.78)**	**0.002**	**0.019**
		CC	56 (30.43)	46 (17.69)	1.44 (0.83–2.51)	0.194	0.354
		T	196 (53.26)	284 (54.62)	Reference		
		C	172 (46.74)	236 (45.38)	1.15 (0.86–1.53)	0.347	0.515
		Dominant			0.72 (0.46–1.11)	0.131	0.265
		Recessive			**2.19 (1.35–3.55)**	**0.001**	**0.012**
*P* value for HWE		0.059					

a adj-ORs were odds ratios adjusted for age, gender, smoking and drinking status.

b *Q* value from Benjamini-Hochberg method for false discovery rate (FDR).

c The dominant model: comparing the combination of heterozygotes and minor allele homozygotes with the major allele homozygotes.

d The recessive model: comparing minor allele homozygotes with the combination of heterozygotes and major allele homozygotes.

### APEX1, MUTYH, XRCC1, XRCC2 and XRCC3 haplotypes

We further analyzed the distribution of haplotypes in cases and controls. All the frequencies of haplotypes are greater than 3% (Table 3). Four haplotypes were constructed in *APEX1* based on the two tagSNPs (rs3136817 and rs1130409), and haplotype GT in *APEX1* had a higher frequency in cases than in controls (*P* = 0.003, adjusted OR = 1.56, 95% CI: 1.17–2.09). Six haplotypes were constructed in *MUTYH* based on the three tagSNPs (rs3219493, rs3219472 and rs3219476). Haplotype CCC in *MUTYH* was the most frequent haplotype in cases and in controls (44.5%), and the frequency of this haplotype was lower in patients compared to healthy controls (*P* = 0.004, adjusted OR = 0.65, 95% CI: 0.49–0.87). Ten haplotypes were constructed in *XRCC1* based on the five tagSNPs (rs1799782, rs2023614, rs2293036, rs3213356 and rs25487). Haplotype GGGTC in *XRCC1* had a higher frequency in cases than in controls (*P* = 0.002, adjusted OR = 2.05, 95% CI: 1.30–2.24), whereas haplotypes GCGCC and GCGTT in *XRCC1* had lower frequencies in cases than controls (*P* = 0.001, adjusted OR = 0.40, 95% CI: 0.23–0.71; *P* = 0.005, adjusted OR = 0.60, 95% CI: 0.42–0.86, respectively). Ten haplotypes were constructed in *XRCC3* based on the five tagSNPs (rs1799794, rs861534, rs861537, rs861531 and rs861530), and haplotype TTTAT had a lower frequency in cases than controls (*P* = 0.009, adjusted OR = 0.36, 95% CI: 0.16–0.80). These associations were remained significant after FDR correction. However, no significant differences were found for the haplotypes in *XRCC2*.

**Table 3:** Frequency distributions of haplotypes of DNA repair genes in cases and controls

Gene	Haplotype	Frequency	Cases	Controls	Adj-OR (95% CI)^a^	*P* value	*Q*_FDR_^b^
APEX1^c^	GC	0.128	0.099	0.156	0.60 (0.39–0.92)	0.018	0.054
	GT	0.342	0.391	0.292	**1.56 (1.17–2.09)**	**0.003**	**0.021**
	TC	0.034	0.030	0.038	0.79 (0.36–1.72)	0.549	0.769
	TT	0.497	0.479	0.515	0.97 (0.66–1.14)	0.311	0.502
MUTYH^d^	CCC	0.445	0.386	0.503	**0.65 (0.49–0.87)**	**0.004**	**0.021**
	GCA	0.148	0.140	0.156	0.91 (0.61–1.37)	0.653	0.807
XRCC1^e^	ACATC	0.228	0.188	0.267	0.74 (0.52–1.05)	0.093	0.195
	GCGCC	0.094	0.052	0.135	**0.40 (0.23–0.71)**	**0.001**	**0.021**
	GCGTC	0.222	0.243	0.201	1.51 (1.07–2.14)	0.019	0.050
	GCGTT	0.233	0.175	0.291	**0.60 (0.42–0.86)**	**0.005**	**0.021**
	GGGTC	0.111	0.139	0.083	**2.05 (1.30–2.24)**	**0.002**	**0.021**
XRCC2^f^	AA	0.062	0.051	0.072	0.69 (0.37–1.29)	0.238	0.417
	AT	0.794	0.803	0.785	1.08 (0.75–1.56)	0.676	0.789
	CT	0.131	0.136	0.126	1.09 (0.71–1.66)	0.708	0.743
XRCC3^g^	CCCAT	0.034	0.029	0.039	0.78 (0.33–1.83)	0.564	0.740
	CCCCC	0.033	0.034	0.031	1.18 (0.52–2.71)	0.694	0.767
	CCCCT	0.349	0.308	0.389	0.77 (0.56–1.06)	0.111	0.212
	TCCCT	0.113	0.134	0.091	1.68 (1.05–2.68)	0.028	0.065
	TCTAC	0.037	0.035	0.039	0.96 (0.43–2.13)	0.921	0.921
	TCTCC	0.255	0.231	0.278	0.85 (0.60–1.21)	0.367	0.551
	TTTAT	0.054	0.028	0.079	**0.36 (0.16–0.80)**	**0.009**	**0.032**

a adj-ORs were odds ratios adjusted for age, gender, smoking and drinking status.

b *Q* value from Benjamini-Hochberg method for false discovery rate (FDR).

c The order of SNPs in APEX1 is rs1130409, rs3136817.

d The order of SNPs in MUTYH is rs3219493, rs3219472, rs3219476.

e The order of SNPs in XRCC1 is rs1799782, rs2023614, rs2293036, rs3213356, rs25487.

f The order of SNPs in XRCC2 is rs3218408, rs3218454.

g The order of SNPs in XRCC3 is rs1799794, rs861534, rs861537, rs861531, rs861530.

### SNP-SNP interactions analysis

It has been reported that the interactions of different SNPs can magnify the effect magnitude of individual SNPs [24]. Thus, we asked whether there is any interaction among the candidate SNPs of DNA repair genes in the risk of bladder cancer. We first performed MDR, a data-mining analytical approach to find a best model by testing the accuracy and cross-validation consistency. The results are shown in Table 4. The model consisting of two loci of *XRCC3* rs1799794 and rs861530 in HRR genes turned out as the best MDR model. This model had the maximal testing accuracy of 0.701 and the maximal cross-validation consistency of 10 out of 10 (*P* value = 0.001; *Q*_FDR_ = 0.007). However, for twelve polymorphisms of five genes in BER genes, no best model of interaction was found (data not shown).

**Table 4:** MDR models of seven SNPs of XRCC2 and XRCC3 gene in cancer cases and controls

MDR Models^a^	Testing balance accuracy	CVC^b^	*P* value	*Q*_FDR_^c^
rs861530	0.682	10/10	0.063	0.110
rs1799794 rs861530	**0.701**	**10/10**	0.001*	**0.007**
rs1799794 rs3218454 rs861531	0.691	10/10	0.058	0.135
rs1799794 rs861537 rs861531 rs861530	0.697	10/10	0.037	0.129
rs3218454 rs1799794 rs861537 rs861531 rs861530	0.688	10/10	0.072	1.101
rs3218408 rs3218454rs1799794 rs861537 rs861531 rs861530	0.684	9/10	0.272	0.272
rs3218408 rs3218454 rs1799794 rs861534 rs861537 rs861531 rs861530	0.676	10/10	0.101	0.118

a MDR, multifactor dimensionality reduction.

b CVC, cross-validation consistency.

c *Q* value from Benjamini-Hochberg method for false discovery rate (FDR).

* The overall best MDR model.

**Figure 1:**
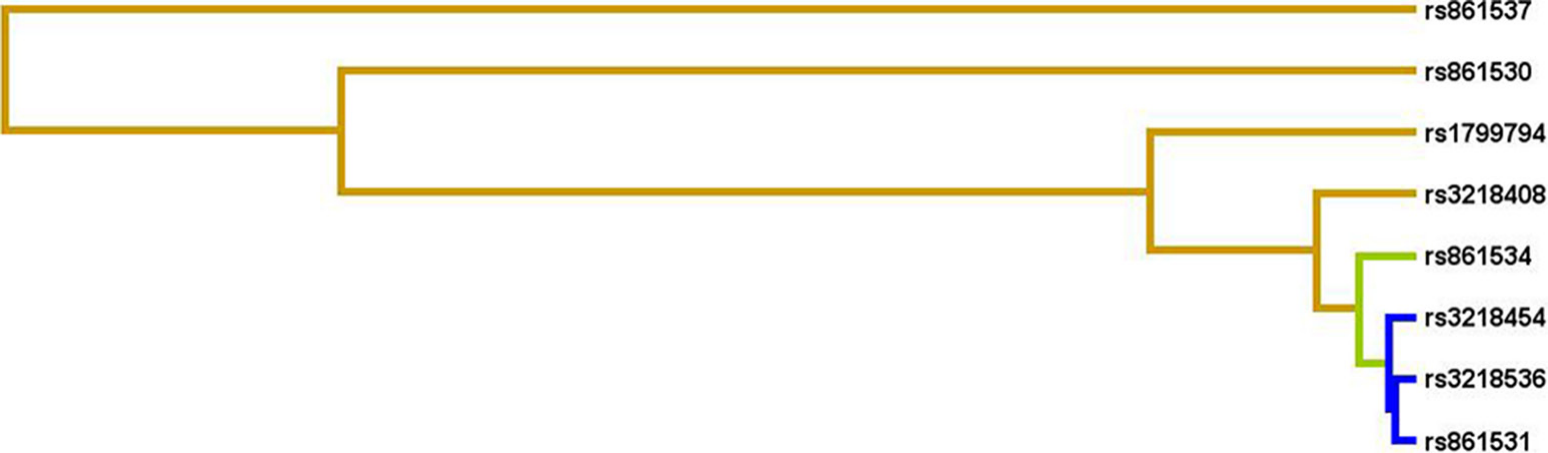
MDR dendrogram for SNP-SNP interactions The colors of lines (from tan to green to blue) indicate the decreased strength of synergistic interaction.

The dendrograms provided by MDR were examined to assist in the visualization and interpretation of potential interactions [25]. MDR produced the interaction dendrogram for the SNP epistasis models across all genes. Dendrogram illustrated how various clusters of SNPs exhibited synergistic interaction (tan lines), weak synergistic interaction (green lines) or redundancy (blue lines). As observed in the dendrogram (Figure 1), the rs1799794, rs3218408, rs861534, rs3218454 and rs861531 belonged to one cluster, while rs861530 belonged to another cluster. The rs1799794 and rs861530 of two clusters showed synergistic interaction, while rs861537 showed a synergistic association with both the clusters. However, every other combination of the interaction provided weak synergistic interaction or redundant information. Redundancy refers to the situation in which the entropy-based interaction between two SNPs provides less information than the entropy-based correlation between the pair.

## DISCUSSION

In this study, we investigated the associations of nineteen polymorphisms of seven DNA repair genes with the risk of bladder cancer among 487 Han Chinese using a tagSNP-based approach. Previous molecular epidemiological studies in different populations have shown the associations between individual genetic variants and bladder cancer risk [4, 26]. To the best of our knowledge, this is the first report assessing the association of the SNPs in multiple DNA repair genes both individually and interactively with bladder cancer risk in the Han population of Northwest China. Moreover, most polymorphisms in our study have not been evaluated previously in bladder cancer.

Polymorphisms in the APEX nuclease (multifunctional DNA repair enzyme) 1 gene (*APEX1*) may be involved in the carcinogenesis by failue to correct DNA damage [27]. Our results suggest that *APEX1* rs3136817 TC genotype and C allele were associated with decreased risk of bladder cancer. *APEX1* rs3136817 is located in intron region. Functional intronic SNPs have been shown to alter RNA secondary structure, thereby influencing mRNA splicing and translation [11]. Thus, we speculate that this polymorphism might regulate *APEX1* mRNA processing and translation. Using the internet-based computer-modeling program mfold [28], we found that there are differences in the predicted RNA secondary structure between the wild-type and mutant homozygotes (Figure S1A and S1B). The mutant CC genotype had a structure with a Δ*G* of −0.20, while the wild-type TT had a Δ*G* of −0.60, suggesting that the RNA secondary structure of the wild-type is more stable. In addition, the shift of stem and loop position may affect the splicing rate of RNA via impacting the formation of a spliceosome due to bringing important splicing signals closer together in one than another, or un/masking cryptic splice sequences [29, 30].

Human MutY glycosylase homolog (MUTYH) is specifically involved in the removal of adenines mismatched with 8-OHdG resulting from DNA replication errors and DNA recombination [31]. Although *MUTYH* rs3219472 polymorphism has been identified in cholangiocarcinoma and esophageal cancer [17, 18], there have been no reports on the *MUTYH* rs3219472 and rs3219493 variants and the susceptibility to bladder cancer. In the present study, we observed that *MUTYH* rs3219493 GG genotype and G allele had an increased risk of bladder cancer, and the finding is in consistent with the previous studies in other types of cancer [15, 16].

X-ray repair cross-complementing group 1 (XRCC1) is involved in the repair of DNA base damage and single-strand DNA breaks by binding DNA ligase III at its carboxyl and DNA polymerase β and poly (ADP-ribose) polymerase at the site of the damaged DNA [32]. Polymorphisms in *XRCC1* have been demonstrated to associate with DNA adduct formation and an increased risk of cancer development [33]. Our study is the first report showing that *XRCC1* gene rs3213356 and rs25487 CT genotype decreased the risk of bladder cancer, whereas *XRCC1* rs3213356 CC and rs1799782 AA genotypes increased the risk of the disease in Chinese population. These susceptible intronic SNPs (rs3113356 and rs2023614) may be functional as others, which have been shown to induce aberrant splicing via the disruption of splicing enhancers and alteration of the pre-mRNA and further impair the translation efficiency [34, 35]. *XRCC1* rs1799782 is an exonic SNP with a missense change of Arg194Trp substitution. It has been shown that missense SNPs may also affect mRNA stability, translational kinetics and splicing, resulting in the alteration of both structure and abundance of protein and its functions [36]. However, our study is not in agreement with the previous studies reported by Wu et al., who showed no significant association existing between *XRCC1* rs1799782 and bladder cancer in an American population [37], and by Stern et al, who did not find the association in non-Latino white population [38]. The discrepancy between the previous studies and ours may be most likely due to ethnicity from different population, indicating the role of genetic factors in the susceptibility to the disease.

The X-ray repair cross-complementing group 3 (XRCC3) is a member of an emerging family of Rad-51-related proteins, and take part in homologous recombination to repair DSBs and maintain integrity of the genome [39]. In this study, we found individuals with *XRCC3* gene rs1799794 CT, rs861531 AC and rs861530 CT genotypes had a decreased risk of bladder cancer, indicating that the heterozygotes of *XRCC3* gene rs1799794, rs861531 and rs861530 are protective genotypes. As *XRCC3* rs1799794 is located in the 5′ UTR region, the underlying mechanism of its function may be related to its alteration in local DNA secondary structure or functional motif, thereby affecting the binding affinities of the relevant transcription factors [40].

Previous studies have reported that some haplotypes of DNA repair genes associated with cancer risk [41–43]. Our study showed that GGGTC and GT haplotypes increased the risk of bladder cancer, whereas GCGCC, GCGTT, CCC and TTTAT haplotypes decreased the risk. Take together, the joint effect of genetic variants may result in more significant alteration of DNA repair capacity compared to a single locus.

We also applied MDR, a promising data-mining approach for overcoming some limitations of traditional parametric statistics such as logistic regression [22, 23] particularly in the case-control studies with a relatively small sample size [44, 45], to detect and characterize high-order gene-gene interactions. The application of MDR in our study to the bladder cancer case-control dataset identified the statistically significant two-loci best model from seven DNA repair genes. It is not surprising to find that the two polymorphisms in the overall best model were also statistically significant in our single-locus analysis. Moreover, our haplotype analysis also found that the estimated frequencies of TTTAT haplotype of *XRCC3* were consistently higher in controls than cases, indicating the interactive role of these two polymorphisms in combination (carrying rs1799794-T and rs861530-T alleles) was particularly evident in the protection from bladder cancer. Even though MDR is a useful method for identifying epistasis, the power of MDR in the presence of noise that is common to many epidemiological studies is unknowable. Moreover, the possible existence of residual confounding from the incompletely measured or unmeasured physiologic covariates cannot be excluded.

The major limitation of our study is the relatively small sample size besides the common issue of misclassification and recall bias for lifestyle factors in case-control studies. As such, a chance cannot be ruled out for some of the significant findings. However, we adjusted for multiple comparisons using FDR correction due to the number of SNPs examined in the study. Finally, because our study subjects were entirely of Han Chinese ancestry, the confounding from ethnicity has been limited. In contrast, this will limit the generalizability of our findings to other ethnic populations in Chinese population.

In Conclusion, the variants of rs3136817, rs3219493, rs3213356, rs25487, rs1799782, rs1799794, rs861531 and rs861530 as well as some haplotypes are significantly associated with the risk of bladder cancer. Moreover, our findings provide evidence that *XRCC3* gene rs1799794 and rs861530 might exert both independent and interactive effects on the bladder cancer. As bladder cancer is a multifactorial complex disorder, large well-designed longitudinal studies attempting to elucidated high-order gene-gene and gene-environment interactions, as well as *in-vitro* and *in-vivo* studies for biological functions of DNA repair genes, are required in future investigation in the susceptibility to bladder cancer.

## MATERIALS AND METHODS

### Patients and controls

Between May, 2007 and May, 2013, a total of 227 patients with bladder cancer and 260 age-matched healthy volunteers from the Second Hospital of Lanzhou University (Lanzhou, China) were enrolled in this study. All cases reside in Gansu province and were histopathologically confirmed and staged according to the tumor-node-metastasis staging system of the Union for International Cancer Control. Exclusion criteria included metastasized cancer from other organs and previous radiotherapy or chemotherapy. The tumors were classified according to the 2016 WHO classification [19]. Pathology slides (or tissue blocks) from all patients with urothelial carcinoma were obtained from the original pathology departments and confirmed by two independent pathologists. Information regarding gender, age, cigarette smoking and alcohol consumption were obtained from medical records. The controls were frequency-matched to cases by age, region and ethnicity, and were randomly selected volunteers without any personal cancer history, family bladder cancer history and other medical situation during routine medical fitness examination. Two classifications (ever, or no) are used in defining smoking and drink. A written informed consent was obtained from each patient and this study was approved by the Second Hospital of Lanzhou University ethical review board.

### SNP selection and genotyping

All SNPs in the DNA repair-associated candidate genes were selected from HapMap CHB database (phase 2, build 35) (http://www.hapmap.org) using the Tagger program implemented in Haploview version 4.1. The tag-SNPs were chosen based on the following criteria: a) minor allele frequency (MAF) ≥ 10%; b) only 1 SNP should be selected within the same LD block defined as pair-wiser *r*^2^ ≥ 0.8; c) For each gene, spanning 2 kb upstream of the 5′ end and 1 kb downstream of the 3′ end. The seven DNA repair candidate genes were *PARP1* gene (rs1805415), *OGG1* gene (rs2072668), *APEX1* gene (rs3136817 and rs1130409), *MUTYH* gene (rs3219493, rs3219476 and rs3219472), *XRCC1* gene (rs3213356, rs25487, rs2293036, rs2023614 and rs1799782), *XRCC2* gene (rs3218408 and rs3218454), and *XRCC3* gene (rs1799794, rs861537, rs861534, rs861531 and rs861530).

In patient group, pathologically confirmed paraffin-embedded paracancerous tissues (resected 2.5 cm away from the tumor edge) with H&E-staining by two independent pathologists were used for genomic DNA extraction using Miobio DNA kit magnetic (Miobio, China). In control group, five ml of venous blood specimens were collected in tubes containing EDTA for genomic DNA extraction using the universal genomic DNA Extraction Kit VER.3.0 (Biotech, China). Genotyping was performed using the QuantStudio™ 12K Flex Real-Time PCR System (Applied Biosystems, Foster City, CA, USA) with a chip-based TaqMan genotyping technology. Genotype data analysis was performed by using OpenArray SNP Genotyping Analysis Software V.1.0.3 (Applied Biosystems). For quality control, genotyping was done with the blind on subject status, and 10% of cases and controls were randomly selected and genotyped twice by different individuals, and the reproducibility was 100%. Additionally, 5% samples of each SNP were randomly selected and confirmed by direct sequencing, the reproducibility of both was 100%.

### Statistical analysis

Deviations of observed genotype frequencies of SNPs were tested for Hardy-Weinberg equilibrium (HWE). Associations between nineteen SNPs and bladder cancer risk were estimated by odds ratios (ORs) and their 95% confidence intervals (CIs) using unconditional logistic regression with adjustment for age, gender, smoking and drinking status in different genetic models (codominant, dominant, recessive, and additive models) as previously described [20].

We used the SHEsis online software (http://analysis.bio-x.cn/myanalysis.php) [21] to calculate the frequency distributions of all haplotypes. Haplotype frequencies were estimated by the maximum likelihood approach and frequencies > 3% in both cases and controls were examined.

We employed a promising data-mining open-source approach multifactor dimensionality reduction (MDR) (version 3.0, http://www.epistasis.org) to explore the potential nonlinear interactions of multiple polymorphisms of DNA repair genes [22, 23]. MDR is a novel and powerful statistical method for the detection of the overall best combinations of all quantities, which differentiates cases from controls with a maximal sensitivity and specificity, classifying them as high- and low-risk groups. The genotypes of each SNP were coded numerically as 0, 1, and 2. The accuracy of each best model was evaluated by a Bayes classifier in the context of 10-fold cross-validation. A single best model has the maximal testing accuracy and cross-validation consistency simultaneously.

The false discovery rate (FDR) method was used to adjust for the multiple comparisons. An FDR of 0.05 was used as a critical value to assess whether Q_FDR_ value was significant. The statistical analyses were carried out using SPSS 16.0 software.

## SUPPLEMENTARY MATERIALS FIGURE


