# Supplementary material for: Association of nineteen polymorphisms from seven DNA repair genes and the risk for bladder cancer in Gansu province of China
Source: Oncotarget. 2016 May 2;7(21):31372–83. doi: 10.18632/oncotarget.9146 (PMC5058763; doi:10.18632/oncotarget.9146)
Supplement: Supplementary file 1 [file oncotarget-07-31372-s001.pdf]

Association of nineteen polymorphisms from seven DNA repair genes and the risk for bladder cancer in Gansu province of China

Supplementary Materials

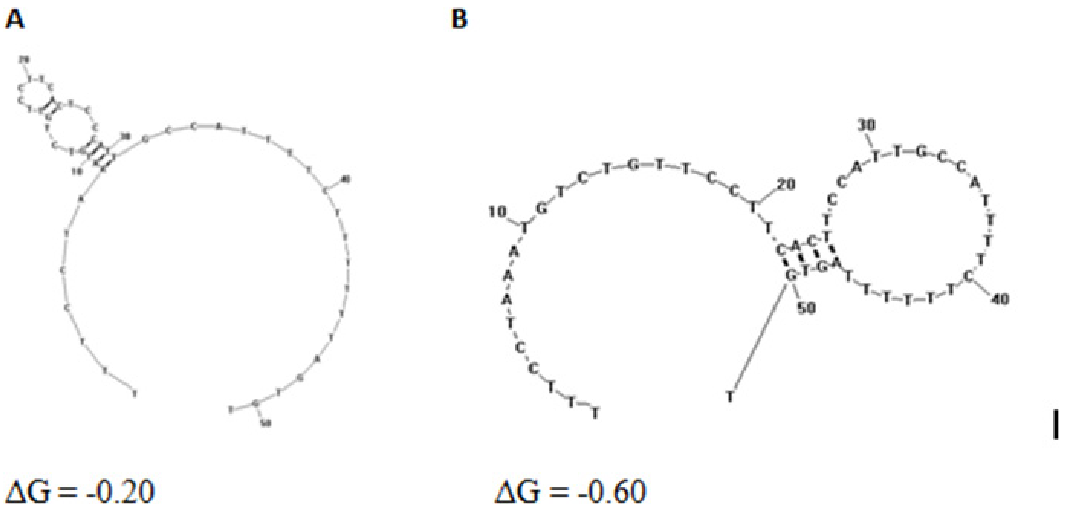

Supplementary Figure S1: Theoretically predicted RNA secondary structures in the region at rs3136817 are shown for the (A) mutant genotype TT and (B) the wild-type CC.
